# The First Report of Environmental Exposure to Barium in 10 Localities Close to Industrial Areas and Ports in the Amazon

**DOI:** 10.3390/ijerph22010109

**Published:** 2025-01-15

**Authors:** Brenda Rodrigues Chagas, Volney de Magalhães Câmara, Karytta Sousa Naka, Thaís Karolina Lisboa de Queiroz, Lorena de Cássia dos Santos Mendes, Iracina Maura de Jesus, Marcelo de Oliveira Lima, Armando Meyer

**Affiliations:** 1 Programa de Pós-Graduação em Saúde Coletiva, Instituto de Estudos em Saúde Coletiva, Universidade Federal do Rio de Janeiro (IESC/UFRJ), Rio de Janeiro 21941-901, Brazil; volney@iesc.ufrj.br (V.d.M.C.); karyttasousa@hotmail.com (K.S.N.); queiroztklq@gmail.com (T.K.L.d.Q.); armando@iesc.ufrj.br (A.M.); 2 Seção de Meio Ambiente (SAAMB), Instituto Evandro Chagas, Secretaria de Vigilância em Saúde e Ambiente, Ministério da Saúde (IEC/SVSA/MS), Ananindeua 67030-000, Brazil; lorenacsmendes1@gmail.com (L.d.C.d.S.M.); iracinajesus@iec.gov.br (I.M.d.J.); 3 Programa de Pós-Graduação em Epidemiologia e Vigilância em Saúde (PPGEVS/IEC/SVSA/MS), Instituto Evandro Chagas (IEC), Ananindeua 67030-000, Brazil; 4 Associação Brasileira de Saúde Coletiva (ABRASCO), Rio de Janeiro 21040-900, Brazil; marcelolima622@gmail.com

**Keywords:** barium, Barcarena, environmental exposure, Amazon

## Abstract

Environmental exposure to metallic contaminants such as barium (Ba) is a worldwide concern, as these metals can even be toxic to the human body. Data on different sources of exposure to Ba and possible routes of entry are important for preventing adverse health effects. Blood Ba levels were evaluated in 10 localities in the cities of Barcarena and Abaetetuba in the Amazon. Ba levels were quantified using induced coupled plasma mass spectrometry, and the data were stratified per epidemiological variables and lifestyle habits. The localities were divided into two groups: Group 1, localities wherein individuals had the lowest median levels (0.299–1.330 µg·L^−1^), and Group 2, localities wherein individuals had the highest median levels (8740–37,300 µg·L^−1^). Factors such as duration of residency, sex, age, smoking status, and alcohol consumption significantly contributed to the increase in exposure. The highest concentrations were associated with drinking water sources such as underground wells and local rivers, as well as the consumption of fish. This is the first study to record Ba exposure in individuals living in localities close to the industrial areas in the Amazon. These findings may facilitate the development of new health surveillance policies and the implementation of preventive measures.

## 1. Introduction

Industrial processes can release metallic elements into the environment in the form of ions or volatile compounds. Losses or inefficiencies in industrial processes result in the emission of atmospheric pollutants or the production of hazardous waste. Depending on the physical and chemical characteristics and the mobilization capacity of these contaminants, occupational or environmental exposure of humans to them may occur [1,2,3,4].

In the 1980s, large multinationals began industrial production activities in the city of Barcarena, Brazil, and were responsible for the release of pollutants, including toxic metals, and numerous environmental disasters in the area, such as contamination of surface and underground water bodies by effluents from kaolin processing; fish deaths; excess soot from industrial chimneys; rupture of pipelines with acidic effluents, reaching rivers; and leakage of waste from kaolin and bauxite processes [5].

These changes have modified ecosystems and increased the health risks to urban and traditional human populations (riverside communities, quilombolas, and indigenous peoples) living in this region [5].

Depending on the chemical composition of the raw materials or substances used during the industrial processing stages, these environmental impacts have resulted in the release of metallic contaminants into the environment, such as barium (Ba). Ba can be emitted through anthropogenic activities, such as the extraction and processing of minerals; data on the chemical composition of effluents generated by industrial processes in Barcarena have shown the presence of this metal in effluents discharged directly into the region’s rivers [3,6,7,8,9]. Ba is a biologically non-essential element and can accumulate through the food chain [10].

The main route of continued absorption of Ba would be through the consumption of contaminated water or food [11,12]. In the human body, this metal can cause blockages in potassium channels, a phenomenon known as hypokalemia, leading to metabolic disorders that cause damage to the cardiovascular, renal, and neurological systems. Ba may also be associated with a high incidence of Multiple Sclerosis [11,12,13].

In Barcarena, urban and traditional populations use river water for human consumption, recreation, and/or subsistence fishing. Considering these habits and the information indicating possible anthropogenic sources of exposure to Ba, toxicological analyses were performed, and the results were cross-referenced with epidemiological and lifestyle data to assess environmental exposure to this contaminant in individuals living in nine locations in Barcarena and one in Abaetetuba, a neighboring city of Barcarena.

## 2. Materials and Methods

### 2.1. Study Design

**Figure 1:**
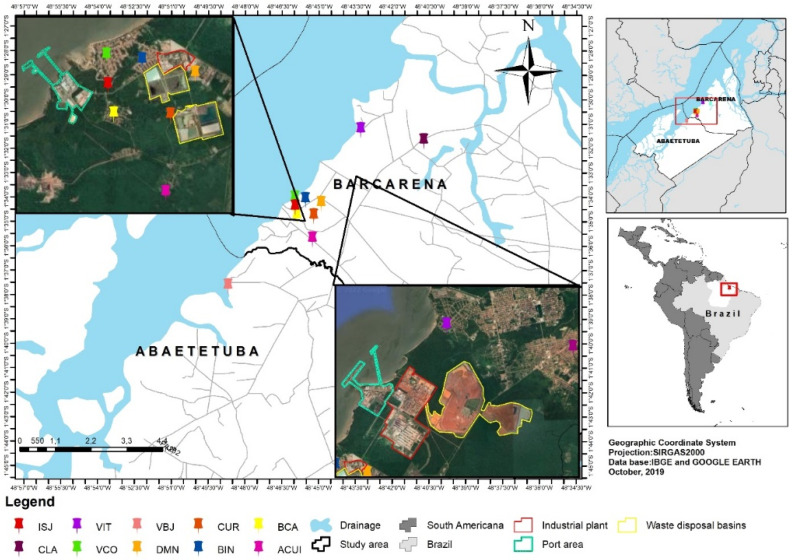
The map of the studied districts.

This was an epidemiological and cross-sectional study for quantifying blood Ba levels in populations exposed to industrial pollutants in nine localities in Barcarena, namely, Acuí (ACUI), Bairro Industrial (BIN), Vila de Itupanema (VIT), Comunidade Laranjal (CLA), Bairro Canaã (BCA), Vila do Conde (VCO), Dom Manoel (DMN), Curuperê (CUR), Ilha São João (ISJ), and one from Abaetetuba known as Vila de Beja (VBJ) (Figure 1).

The collection of blood samples and epidemiological surveys were conducted through home visits between February 2012 and February 2013. The city of Barcarena has a territory of approximately 1310 km^2^ and is located in the Amazon River Delta region in northern Brazil [14]. In the early 1980s, Barcarena had approximately 20,000 inhabitants (22.37/km^2^), and currently, the population is over 120,000 inhabitants (93.32/km^2^) [15]. Due to the local industrial activity, it is among the cities with the highest economic development indicators, with a per capita income of BRL 39,732.60.

Abaetetuba is a municipality located next to Barcarena, with an estimated area of 1610 km², a population of 141,000 inhabitants (87.61/km²), and a per capita income of BRL 8718.18. The selection of these study areas was motivated by geospatial factors and previous reports of risks to human health from the consumption of water contaminated by toxic metals and inhalation of particulate matter (soot) emitted by industries, as shown in Table 1 [16,17,18,19,20,21,22].

The districts of ACUI (N = 88), BIN (N = 188), VIT (N = 209), DMN (N = 43), CUR (N = 22), ISJ (N = 31), VCO (N = 230), and BCA (N = 75) were located up to 1 km from the site where industrial kaolin processing occurs, where there are reports indicating that there is atmospheric emission of particles from boiler chimneys and possible contamination of groundwater used for human consumption from settling basins of liquid effluents from kaolin processing in these districts.

The districts of CLA (N = 296) and VBJ (N = 295) were selected because these districts are relatively distant (3 to 7 km) from where these anthropogenic activities occur and would be parameters for comparing concentration levels by proximity. During the sampling, a portion of the total population of Barcarena, approximately 20,000 inhabitants, was used to calculate the sample size (Sample N = 1477) sufficient for statistical inference, 95% confidence interval for significance.

### 2.2. Epidemiology

The study was approved by the Ethics Committee of the Instituto Evandro Chagas (IEC) (0010/2009). Participants included in this study were individuals of both sexes who resided in the 10 districts studied. This study involved the application of an epidemiological survey and sampling of biological material (blood). Participation was voluntary, and after all individuals signed the Free and Informed Consent Form (TCLE), an epidemiological form was applied with sociodemographic data, habits, and lifestyle variables (schooling, gender, consumption of alcoholic beverages, and the use of cigarettes, or the habit of dyeing hair). In addition, dietary variables (daily consumption of fruits, vegetables, red meat, fish, and seafood or canned food) and variables related to the morbidities reported by the interviewees were included, type of housing, forms of drinking water treatment (the use of mineral water, boiling, coagulation, use of hypochlorite, filtration or the non-treatment of consumed water) and the origin of the water consumed by the interviewees were also included.

### 2.3. Blood Sampling

Blood samples were collected through venipuncture on the forearm (5 mL) using needles and vacuum tubes containing high-purity K2EDTA anticoagulant, indicated for metal analysis. After collection, the samples were immediately transported in thermal boxes. Following this, after thawing and homogenizing the samples, 200 μL of the whole blood was transferred to 15 mL polytetrafluorethylene (PFA) tubes, and 200 µL of Triton X-100 solution (0.1%) and 1% HNO_3_ were added. Subsequently, the sample solutions were stirred (VORTEX, QUIMIS, Diadema, Brazil) and left to stand for 5 min, following which they were adjusted to 4 mL with 1% HNO3 solution. The solutions were again homogenized and centrifuged at 6000 rpm for 10 min (Z206A, HERMLE, Gosheim, Germany). Finally, the supernatant was transferred to 15-mL PFA tubes [28]. The metals were quantified using an inductively coupled plasma mass spectrometer (ICP-MS, Bruker, Billerica, MA, USA, model 820-MS).

The operating conditions of the ICP-MS were 1400 W radio frequency power, 15 L min^−1^ argon flow, Meinhard nebulizer with 0.98 L min^−1^ nebulization flow, and a cyclonic chamber and Scan mode: peak hopping. All blood samples were analyzed in duplicate, with five readings per sample. The Ba isotope measured was Ba-137, and the concentration in the calibration curve varied between 0.600 and 9.600 µg·L^−1^. The quantification limit for Ba was 0.598 µg·L^−1^, and the detection limit was 0.179 µg·L^−1^. The mean recovery percentage of the reference materials used in the quantification of blood Ba was 80.10% for Whole Blood Lyophilized L-1 and 75.6% for Whole Blood Lyophilized L-2.

The samples were prepared, and readings were performed in a clean room environment (class 1000) to minimize the risk of contamination. All blood samples were analyzed in duplicates, with five readings per sample. As an internal standard, multi-element solutions of yttrium, gallium, lanthanum, rhenium, and rhodium were used at a concentration of 5 µg·L^−1^. The equipment ICP-MS was calibrated using the standard addition method with the multi-element solutions,

### 2.4. Statistical Analysis

The results obtained were organized and stored in Microsoft Excel 2013 spreadsheets. Statistical analysis was performed using the Minitab 17.0 and BioStat 5.2 software. Values below the LOQ were considered to be ½ LOQ, as used in other studies [29,30,31]. The Grubbs test was applied to verify the presence of outliers, and these were excluded. Ba concentrations were described using the arithmetic mean (AM), geometric mean (GM), and median (MED). The distribution of normality of variables by groups was determined using the Kolmogorov–Smirnov test (K–S).

The variables studied were sex, age group (>10, 10 to 19, 20 to 59, and ≥60 years), duration of residency in the study area (2 to 5, 6 to 9, and ≥10 years), drinking water source (well or general network and river), alcohol consumption (consumes or does not consume alcohol), smoking (smoker or nonsmoker), and eating habits related to food items (consumption of meat, chicken, fish, seafood, Brazil nuts, vegetables, and fruits), as they could be associated with blood Ba levels.

To compare blood Ba levels among two or more groups, an analysis of variance using the non-parametric Mann–Whitney and Kruskal–Wallis tests was performed. Dunn’s test was also used as a post-hoc test to identify the pairs different from samples with more than three groups.

To verify possible associations between Ba contents and predictive variables, Spearman’s Correlation Test was used. Variables that showed a significant correlation with Ba levels in the sequence were evaluated by simple regression analysis. The concentration of Ba was defined as a dependent variable, with the independent variables: proximity to impacted rivers (next door, near, and far), proximity to the kaolin industry (next door, near, and far), water consumption (deep wells, wells shallow, and river water consumption), and frequency of weekly fish consumption (<1 time, 2 to 4 times, and <4 times).

All tests were performed using a 95% confidence interval and a *p*-value of 0.05 for significance.

## 3. Results

The study involved 1477 individuals. Table 2 shows the general characteristics of the population assessed from each locality. Most individuals were women (54.91%), adults aged 20–59 years (47.33%), and had lived in the area for >5 years (81.04%). The location with the highest number of individuals evaluated was CMA (N = 296), and the one with the lowest participation was CUR (N = 22).

The K–S normality test for blood Ba levels of all evaluated individuals showed a skewed distribution (KS = 0.290, *p* < 0.010). Table 3 shows the AM, MED, GM, standard deviation, and percentage distributions of blood Ba levels for all localities. The results showed differences in the median concentration ranges among the localities, based on which they were divided into two distinct groups: Group 1, localities wherein the median blood Ba levels ranged from 0.299 to 1.330 μg·L^−1^ (ACUI, BIN, VIT, CLA, and VCO) and Group 2, localities wherein the median blood Ba levels ranged from 9.570 to 37.300 μg·L^−1^ (BCA, VBJ, DMN, CUR, and ISJ).

The Kruskal–Wallis test demonstrated that there were significant differences (H = 567.29, *p* = 0.000) in the median blood Ba levels among the 10 localities evaluated. In addition, Dunn’s test was used to determine which pairs of localities had similar median values (Table 4). These results showed that there were no significant differences in the median blood Ba levels between BIN and ACUI (*p* = 0.549) or between CLA and VCO (*p* = 0.918), with both pairs belonging to Group 1.

There were also no significant differences between the medians of BCA and DMN (*p* = 0.129), VBJ and DMN (*p* = 0.894), VBJ and CUR (*p* = 0.338), DMN and CUR (*p* = 0.469), and CUR and ISJ (*p* = 0.241). All localities in the second group had higher blood Ba levels. All individuals with the lowest median blood Ba concentrations resided in locations that were different from the locations of individuals with the highest concentrations, confirming that the categorization of groups was appropriate.

The Grubbs outlier test was performed to determine outliers for each location. The blood Ba level of each outlier, results of the Grubbs test, and the level of significance were as follows: ACUI (4.867 µg·L^−1^; G = 4.61, *p* > 0.001), BIN (50.451 µg·L^−1^, G = 13.07, *p* > 0.001), VIT (46.606 µg·L^−1^; G = 6.04; *p* > 0.001), CLA (113.698 µg·L^−1^; G = 5.44, *p* > 0.001), VCO (27.033 µg·L^−1^; G = 5.67; *p* > 0.001), BCA (84.266 µg·L^−1^; G = 5.32, *p* > 0.001), VBJ (55.653 µg·L^−1^; G = 4.71, *p* > 0.001), DMN (21.131 µg·L^−1^; G = 4.47; *p* > 0.001), CUR (62.269 µg·L^−1^; G = 3.26; *p* > 0.001), and ISJ (135.850 µg·L^−1^; G = 3.33; *p* > 0.001). After determining the outliers, these were removed to perform the statistical analysis for each locality.

**Figure 2:**
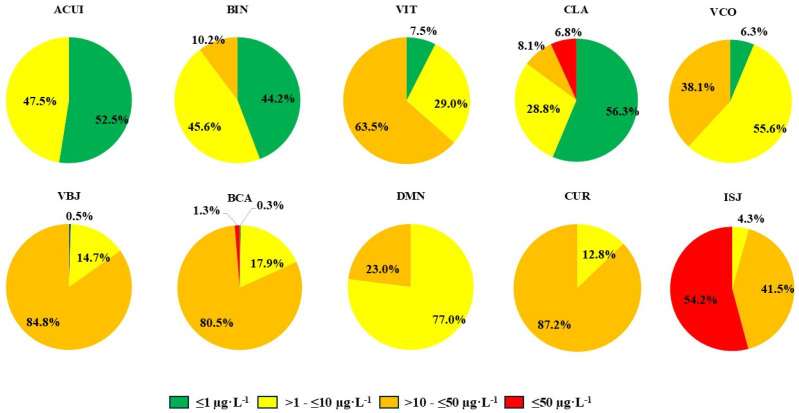
The frequency of distribution of blood Ba levels (µg·L^−1^) by the communities evaluated.

Figure 2 shows the frequency of distribution of blood Ba levels over four concentration ranges: ≤1 µg·L^−1^, >1 to ≤10 µg·L^−1^, >10 to ≥50 µg·L^−1^, and >50 µg·L^−1^. The distribution frequencies of the blood Ba levels were different among the ranges mentioned above, based on which the 10 localities could be divided into two different groups: Group 1 showed a frequency of Ba blood levels with the lowest concentrations < 1 µg·L^−1^, mainly in ACUI and BIN, with concentrations ranging from >1 to ≤10 µg·L^−1^ in VIT, CLA, and VCO. In Group 2 (BCA, VBJ, DMN, CUR, and ISJ), the frequency with the highest concentrations for blood Ba levels ranged from >10 to ≥50 µg·L^−1^, with concentrations reaching > 50 µg·L^−1^.

Table 5 and Table 6 show data for all districts with Ba concentrations stratified per the co-variables studied (sex, age, duration of residency in the study area, alcohol consumption, smoking status, and source of drinking water).

**Figure 3:**
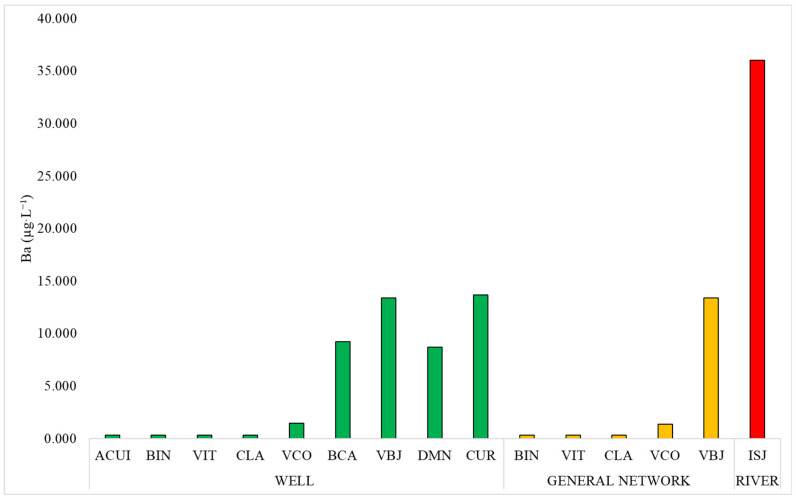
The concentration of Ba (µg·L^−1^) in blood by drinking water sources.

The results showed significant differences among different sources of drinking water in BIN (*p* = 0.002), between male and female residents in CUR (*p* = 0.009), and among age groups in DMN (*p* = 0.041). Figure 3 depicts the trend in water consumption.

Table 7 and Table 8 show the data for all locations with Ba concentrations stratified per eating habits. Significant differences were found in individuals from VIT who consumed Brazil nuts (*p* = 0.035); individuals in CLA who consumed fish (*p* = 0.034); individuals from VCO who consumed meat (*p* = 0.050), fish (*p* = 0.020), seafood (*p* = 0.010), vegetables (*p* = 0.031), and fruits (*p* = 0.020); and individuals from VBJ who consumed Amazon nuts (*p* = 0.052) and fruits (*p* = 0.018), compared with the individuals who did not consume these food items.

**Figure 4:**
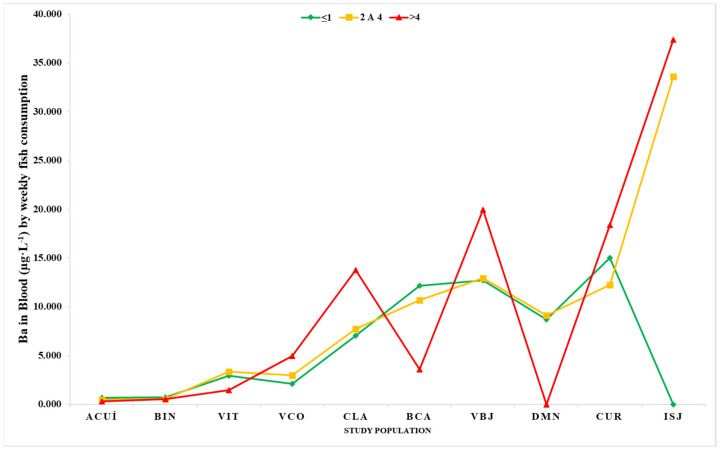
Blood concentrations of Ba (µg·L^−1^) by weekly fish consumption.

Significant differences were found in blood Ba levels between individuals from CLA and VCO who consumed fish more than four times a week and individuals who did not. Figure 4 shows the associations between blood Ba levels and fish consumption for all locations.

**Figure 5:**
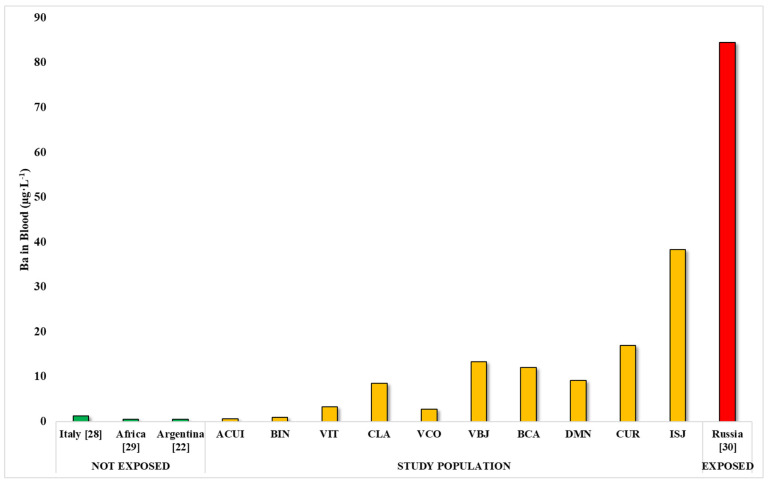
Mean levels of Ba (μg·L^−1^) in exposed and unexposed populations and in the studied population.

It was noted that individuals in 8 of the 10 localities had average blood Ba concentrations above the normal reference values reported in the literature (Figure 5).

Statistical analysis in Table 9 demonstrates the Spearman correlation coefficients and linear regression to identify the influence between Ba and the independent variables, also considering statistical significance (*p* = 0.05). The correlation analysis showed that Ba concentrations in the blood were strongly affected in the vicinity of the kaolin factory by water consumption and the frequency of weekly fish consumption.

## 4. Discussion

This is the first study regarding environmental exposure to Ba in the Amazon in almost 50 years since the beginning of mineral mining and industrial development in the region. Blood is the most frequently used biological sample to measure exposure levels of metals in an organism. However, metal exposure can also be evaluated using other samples such as hair, bones, urine, and feces [9]. Previous studies have shown that the blood levels of metals tend to be higher in populations living close to industrial areas [12,17,32].

When comparing the results of the mean Ba levels in the blood of the study population with the results of studies of unexposed populations, we can observe that 8 of the 10 districts studied (VIT, CLA, VCO, VBJ, BCA, DMN, CUR and ISJ) exhibit higher concentrations than studies of unexposed populations such as Italy (AM = 1.2 µg·L^−1^; N = 590), Argentina (AM = 0.48 µg·L^−1^; N = 31) and Guinea in Africa (AM = 0.43 µg·L^−1^; N = 70) [22,33,34].

However, these levels were lower than those found in a population of mining workers in Russia (AM = 84.5 µg·L^−1^; N = 50) and occupationally exposed individuals [35]. These data indicate that most locations had greater environmental exposure to Ba than that reported in other countries and that living in industrial areas is a factor that contributes to increased blood Ba levels (Figure 5).

Among the localities in Group 1, most individuals from ACUI (91.95%), BIN (88.30%), VIT (71.29%), CLA (57.09%), and VCO (48.70%) had blood Ba levels in the range of normal reference values for environmentally unexposed populations. However, notably, in VIT, CLA, and VCO, a high percentage (28.71–51.30%) of individuals presented with higher concentration ranges, indicating that blood Ba levels of individuals in these three localities may be gradually increasing over time, and if exposure to the source continues, individuals with normal Ba levels may eventually present with the profile of environmentally exposed populations.

These observations correspond with the records of significant increases in industrial production in Barcarena in the last decades [26,36]. The increase in production generates a greater amount of waste, leading to a greater production of effluents and expanding the need for larger areas for waste deposition [3]. The data show that the areas marked for waste deposition have expanded in recent years. Additionally, the distance of these areas from residential localities in the region has gradually decreased [27,36].

In Group 2, most individuals in BCA (79.73%), VBJ (87.46%), DMN (100%), CUR (100%), and ISJ (96.77%) had blood Ba levels higher than the reference range for environmentally unexposed populations. The comparison tests between groups (Table 5 and Table 6) were indicative of a possible relationship between higher blood Ba levels and drinking water sources. Water sources are among the main sources of exposure to metallic elements [32,37].

BIN and VBJ showed blood cadmium (Cd) levels that were 1.5–3 times higher in individuals who consumed well water than in those who consumed general network water and also demonstrated higher blood lead (Pb) concentrations in consumers of well water than in those consuming network water in the DMN locality [17,38].

Ba is known to become naturally bioavailable in the environment from weathering processes, which are normally more intense in tropical regions with high temperatures and humidity [2,8,32,39]. However, Ba can also be introduced into the environment through effluent discharge and waste generated from industrial processes [9,22,40].

Although only the data for BIN indicated a significant association (*p* = 0.002) with drinking water sources, Figure 3 indicates a trend of increases in the median blood Ba levels that were associated with the main sources of drinking water in these localities. Notably, the levels were higher in localities where the main sources of drinking water were wells or rivers. Importantly, ISJ, CUR, and BCA, all in Group 2, are located on the banks of the Curuperê and Dendê rivers, and individuals here presented with higher than normal blood Ba levels.

The Curuperê and Dendê rivers were, for almost a decade, the final destination of untreated effluents and debris from countless dam ruptures in the basins where kaolin processing plants had been constructed close to the spring sources of these water bodies since the 1980s. Data from 2003 and 2007 showed that there was a continuous discharge of untreated effluent on the margins of these water bodies, and the concentrations of Ba in surface waters reached 744 µg·L^−1^ [3,23].

These levels were higher than those found in other rivers in the Amazon, for which surface water levels ranged from 3.1 to 56.1 µg·L^−1^ [41]. They were also lower than the results for drinking water in Santana in Amapá State (56–179 µg·L^−1^), where the drinking water sources were underground wells or rivers (Amazon River) [42]. Studies on the Murucupi River, also located in Barcarena, showed that the Ba levels varied from 14.6 to 76.7 µg·L^−1^, indicating that the levels in the Curuperê and Dendê rivers were higher than the natural levels in the region [18].

Only a study of the population of Kandal, a locality in the Mekong River basin in Cambodia with 1028 µg·L^−1^ of Ba in the drinking water, reported values higher than those in our findings [43]. Therefore, this information indicates that the higher blood Ba concentrations in the ISJ, CUR, and BCA localities may potentially be associated with the use of water from the Curuperê and Dendê rivers, which are water bodies that have received large amounts of effluent discharge and waste for years, without implementation of remediation measures [3].

ISJ is a riverside locality on the banks of the Dendê River, which the population uses for leisure, fishing, and transportation; CUR is located adjacent to the kaolin processing industry and uses the Curuperê River for fishing. The wells in this region are considered to be shallow [23,44]. In cases of man-made disasters such as effluent spills, there are reports of changes in the color and odor of the rivers in these locations, as well as increased fish mortality rates [3,23,25,42].

Owing to the deposition of contaminants in these rivers over the years, the highest levels of Ba in these localities may be associated with particular sources of drinking water and fish consumption. The correlation and regression tests reinforce this assertion: whoever lives next to or close to the kaolin industry and consumes water from shallow wells or rivers is directly linked to having higher concentrations of Ba in the blood. The higher frequency of weekly fish consumption is also directly associated with higher concentrations of Ba in the blood of the population.

Geological studies of the kaolin extracted in the Capim region in the Amazon showed Ba levels between 15.8 and 39.8 mg kg^−1^ in the ores [8,45]. In Botswana, up to 5806 mg kg^−1^ of Ba was found in samples of kaolin deposits [46], and in western Cameroon, there are reports of concentrations up to 699 mg kg^−1^ [47]. Similarly, the chemical profile of trace elements in the bauxite ore mining in Paragominas City, Pará State, showed BA levels in the range of 17 to 70 mg kg^−1^ [41]. Therefore, the presence of high Ba concentrations in the residues and effluents from kaolin and bauxite processing is expected.

The occurrence of the greater availability of Ba in drinking water has been shown in similar situations in Texas, Georgia, New Mexico, Kentucky, and Pennsylvania in the USA [48]. Ingestion of Ba, even for relatively short periods, can cause gastrointestinal disorders, nausea, vomiting, colic, and muscle weakness. Further, ingestion of high levels for a long time can result in cardiovascular, kidney, metabolic, neurological, and mental disorders [11,49,50].

Kaolin is also present in the geological layers of the subsoil in Barcarena and Abaetetuba. Georgia has the second-largest kaolin deposit worldwide [3,45]. However, this study showed that greater proximity to industrial areas and commercial ports contributes to high barium exposure, owing to the impact of large industries on the aquifers and nearby rivers. Among these industrial enterprises, kaolin processing and aluminum production have the greatest environmental impact.

However, BIN, which is also located adjacent to an industrial kaolin processing area, showed the lowest median blood Ba levels (median 0.299 µg·L^−1^ N = 120 for wells; median 0.299 µg·L^−1^ N = 67 for general network). In BIN after 2007, when the kaolin tailing basin ruptured, resulting in effluent contamination of the drinking wells, a deep well was built for water collection and subsequent distribution through the general network. At the time of this study, most residents no longer used shallow wells and did not normally have direct contact with the river [17,51]. Therefore, despite the proximity to the kaolin processing industries, the locality did not have high exposure levels. This finding indicates that the use of deep wells may reduce Ba exposure in the region.

ACUI (MED = 0.299 µg·L^−1^; N = 87) is a locality with an indigenous population that practices rural habits of the local cultivation of fruits and vegetables and animal husbandry in their backyards. However, this locality is situated at some distance from the industrial areas. On the other hand, VIT (MED = 0.299 µg·L^−1^ N = 114 for wells; MED = 0.299 µg·L^−1^ N = 92 for the general network) and CLA (MED = 0.320 µg·L^−1^ N = 215 for wells; MED = 0.300 µg·L^−1^ N = 80 for the general network) are more urbanized localities and less dependent on rivers in the region.

VCO (MED = 1.432 µg·L^−1^ N = 100 for wells; MED = 1.346 µg·L^−1^ N = 125 for the general network) is one of the oldest localities in the region. It is situated on the banks of the Pará River and next to large ports that are used daily for the transport of raw materials and manufactured products [52].

BCA (MED = 9.21 µg·L^−1^ N = 74 for wells) and DMN (MED = 8672 µg·L^−1^ N = 42 for wells) are located close to the kaolin processing industries and record high levels of atmospheric pollution and deposition of untreated effluents or industrial waste. However, most residents of these localities use water from shallow wells [53].

In VBJ (Median 13,381 µg·L^−1^ N = 156 for wells; 13,354 µg·L^−1^ N = 138 for the general network), there were no significant differences in the median Ba levels between individuals who consumed drinking water from wells and those who consumed water from the general network. The local sanitation plan of the locality comprises a small water collection and distribution system. A semi-artesian well supplies water to only 26.66% of the population, whereas the rest use other wells or rivers [54].

Although only data for CLA (*p* = 0.034) and VCO (*p* = 0.031) showed that there was a significant association of Ba levels with the weekly frequency of fish consumption (more than four times a week) (Figure 4), there were significant increments in the blood Ba level in individuals from other localities (Figure 2). Ba is known to accumulate in fish and other aquatic organisms [11,49]. Therefore, in communities with a high consumption of fish, this can be one of the main sources of exposure to toxic metals [13,55]. The sustained higher-than-normal Ba levels in surface waters may be indicative of the accumulation of this metal in the fish population, which can ultimately lead to high Ba levels in the local population.

Data for VIT and VBJ showed significant associations between Ba levels and consumption of Brazil nuts two to four times a week. This finding corroborates reports that show that Ba accumulates in Brazil nuts, fruits, and vegetables [11,13,56,57].

Although most participants were women, no significant associations were observed between blood Ba levels and sex, confirming that blood Ba levels depend mainly on possible sources of exposure relevant to the locality. In Cambodia, there were no significant associations of Ba concentrations in hair with age or sex, corroborating the assumption that these variables do not influence Ba exposure, and the main factor is the proximity of exposure sources [43].

Alcohol consumption and cigarette smoking were not associated with increased blood Ba levels, demonstrating that these habits are not exposure sources. Studies show that blood Ba levels are primarily associated with the proximity of exposure sources. Although Ba is known for its acute toxicity, epidemiological data for chronic exposure are still scarce. There are also important gaps in the current literature with respect to the identification of vulnerable populations and the factors that make some individuals susceptible to Ba toxicity [11,32,40].

We emphasize that the main effect of Environmental Exposure to Barium is associated with changes in the sodium–potassium pump that regulates the circulatory system and may be associated with possible changes in the blood pressure of exposed individuals. This information on blood pressure was not collected during this research, but it alerts health agencies to the need for better monitoring of cardiovascular diseases through a future biomonitoring program. Cardiovascular diseases are the main health indicators associated with exposure to barium.

## 5. Conclusions

This study provided data on the blood Ba levels in people living near the port and industrial areas in the Amazon and showed possible sources of human exposure. The average Ba levels found in individuals from all communities indicated continual exposure in the region; individuals from 8 of the 10 communities had blood concentration levels up to 50 times higher than the normal levels previously reported in the literature.

There is evidence that living near kaolin processing plants and drinking water from rivers directly increases Ba levels in the blood of this population. Populations living on the banks of rivers, such as the Curuperê and Dendê, may be at greater risk of adverse health effects.

In addition, greater exposure to Ba was observed in individuals with higher weekly fish consumption. Fish is part of the diet of riverside and quilombola populations, which may be evidence of the accumulation of Ba in the trophic chain of the fish consumed.

The results of this study provide relevant toxicological information regarding Ba exposure in the Amazon, and the results may be used for comparisons of future epidemiological studies in Brazil and other countries. There is a lack of studies on monitoring and systematic management of environmental exposure to metals in Brazil.

The need for these studies is based on laboratory, clinical, and epidemiological assessments. Therefore, we advise public health systems that these populations need to be monitored continuously through biomonitoring, especially for populations located near industrial production areas, to identify potential sources of environmental exposure.

## Figures and Tables

**Table 1:** The history of environmental disasters in Barcarena.

Year	Event
2003	Fish mortality [23]
2003	Rupture of dams and discharge of untreated red mud effluents [9]
2003	Discharge of untrated kaolin efluentes [24]
2004	Emission of industrial soot [14]
2006	Seaweed bloms [14]
2006	Emission of industrial soot [14]
2006	Dicharge of untread efluentes [14]
2007	Discharge of untreated kaolin effluents [14]
2007	Rupture of dams and discharge of untreated kaolin effluents [25]
2008	Wreck of fuel cargo [14]
2009	Rupture of dams and discharge of untrated red mud effluents [26]
2010	Emission of industrial soot [19]
2011	Discharge of untreated kaolin effluents [14]
2012	Discharge of untreated kaolin effluents [14]
2014	Discharge of untreated kaolin effluents [14]
2015	Wreck of fuel cargo and 5.000 oxen [14]
2016	Discharge of untreated kaolin effluents [21]
2018	Discharge of untrated red mud effluents [27]

**Table 2:** The localities’ characteristics, Barcarena e Abaetetuba Cities, 2012–2013.

LOCALITY	Female		Male		Age Group Years (N)				Residence Time Years (N)			Total
	N	%	N	%	0–10	11–19	20–59	≥60	<2	2–5	>5	
Acuí	49	55.68	39	44.32	20	21	36	11	5	11	72	88
Bairro Industrial	98	52.13	90	47.87	42	43	91	12	18	41	129	188
Vila de Itupanema	122	58.37	87	41.63	44	46	96	23	2	16	191	209
Comunidade Laranjal	167	56.42	129	43.58	47	82	145	22	16	30	250	296
Vila do Conde	133	57.83	97	42.17	36	65	107	22	10	13	207	230
Bairro Canaã	38	50.67	37	49.33	23	11	36	5	6	40	29	75
Vila de Beja	163	55.25	132	44.75	52	59	140	44	14	36	245	295
Dom Manuel	19	44.19	24	55.81	11	9	19	4	6	4	33	43
Curuperê	9	40.91	13	59.09	4	2	15	1	*	1	21	22
Ilha São João	13	41.94	18	58.06	8	7	14	2	8	3	20	31
General total	811	54.91	666	45.09	287	345	699	146	85	195	1197	1477

* Does not present any individual in the studied group.

**Table 3:** Statistical analysis of the blood Ba concentrations (μg·L^−1^) of the studied populations, Barcarena and Abaetetuba, 2012–2013.

LOCALITY	N	AM	Median	GM	SD	Range	P5%	P25%	P75%	P95%
Acuí	88	0.583	0.299	0.382	0.910	0.299–4.867	0.299	0.299	0.299	2.444
Bairro Industrial	188	0.890	0.299	0.424	3.793	0.299–50.451	0.299	0.299	0.299	2.286
Vila de Itupanema	209	3.247	0.299	0.778	7.183	0.299–46.606	0.299	0.299	2.629	17.769
Comunidade Laranjal	296	8.500	0.310	1.329	19.350	0.300–113.700	0.299	0.300	4.390	61.278
Vila do Conde	230	2.763	1.330	1.197	4.282	0.299–27.033	0.299	0.299	3.202	12.118
Bairro Canaã	75	11.980	9.570	5.179	13.590	0.300–84.270	0.299	1.950	15.460	34.078
Vila do Beja	295	13.269	13.542	8.239	9.001	0.299–55.653	0.299	7.394	17.723	28.457
Dom Manuel	43	9.120	8.740	8.944	2.196	7.156–21.131	7.471	7.871	9.786	10.930
Curuperê	22	16.920	14.240	12.106	13.900	1.980–62.300	3.405	4.940	23.720	32.592
Ilha São João	31	38.350	37.300	26.053	29.310	1.200–135.850	5.076	13.890	59.750	77.096

AM: Arithmetic Mean; GM: Geometric mean; SD: Standard deviation.

**Table 4:** The Dunn’s test results with the adjustment of *p*-values after Kruskal–Wallis H test.

	LOCALITY	Group 1					Group 2			
		ACUI	BIN	VIT	CLA	VCO	BCA	VBJ	DMN	CUR
	BIN	0.549	-	-	-	-	-	-	-	-
Group 1	VIT	*p* < 0.05	*p* < 0.05	-	-	-	-	-	-	-
	CLA	*p* < 0.05	*p* < 0.05	*p* < 0.05	-	-	-	-	-	-
	VCO	*p* < 0.05	*p* < 0.05	*p* < 0.05	0.918	-	-	-	-	-
	BCA	*p* < 0.05	*p* < 0.05	*p* < 0.05	*p* < 0.05	*p* < 0.05	-	-	-	-
Group 2	VBJ	*p* < 0.05	*p* < 0.05	*p* < 0.05	*p* < 0.05	*p* < 0.05	*p* < 0.05	-	-	-
	DMN	*p* < 0.05	*p* < 0.05	*p* < 0.05	*p* < 0.05	*p* < 0.05	0.129	0.894	-	-
	CUR	*p* < 0.05	*p* < 0.05	*p* < 0.05	*p* < 0.05	*p* < 0.05	*p* < 0.05	0.338	0.469	-
	ISJ	*p* < 0.05	*p* < 0.05	*p* < 0.05	*p* < 0.05	*p* < 0.05	*p* < 0.05	*p* < 0.05	*p* < 0.05	0.241

**Table 5:** The description of the statistical analysis by localities (Group 1) with lower blood Ba levels (µg·L^−1^) by the epidemiological variables.

GROUPS	ACUI					BIN					VIT					CLA					VCO				
	N	AM	MED	RANGE	*p* **	N	AM	MED	RANGE	*p*	N	AM	MED	RANGE	*p* **	N	AM	MED	RANGE	*p* **	N	AM	MED	RANGE	*p* **
Gender																									
Female	49	0.533	0.299	0.299–4.796	0.949	97	0.715	0.299	0.299–11.848	0.48	121	2.765	0.299	0.299–37.069	0.663	166	7.260	0.300	0.300–95.810	0.676	132	2.608	1.355	0.299–23.138	0.821
Male	38	0.536	0.299	0.299–3.365		90	0.528	0.299	0.299–4.773		87	3.420	0.299	0.299–35.784		129	9.290	0.340	0.300–90.280		97	2.723	1.210	0.299–22.080	
Age group (years)																									
0 a 10	20	0.386	0.299	0.299–2.028	0.392	42	0.550	0.299	0.299–2.935	0.793	44	3.390	0.300	0.30–37.070	0.078	47	10.340	2.030	0.300–90.280	0.403	36	2.722	1.518	0.299–23.138	0.839
11 a 19	21	0.507	0.299	0.299–2.495		42	0.682	0.299	0.299–4.772		45	1.364	0.299	0.299–21.08		82	9.660	0.300	0.300–86.270		64	2.478	1.406	0.299–19.085	
20 a 59	35	0.525	0.299	0.299–3.767		91	0.650	0.299	0.299–11.848		96	3.381	0.299	0.299–32.548		144	6.610	0.300	0.300–95.810		107	2.931	1.371	0.299–22.080	
≥60	11	0.884	0.299	0.299–4.796		12	0.503	0.299	0.299–2.748		23	4.210	0.300	0.300–35.780		22	7.910	2.540	0.300–83.270		22	1.738	1.150	0.299–8.3020	
Residence time (years)																									
<2	5	0.299	0.299	0.299–0.299	0.064	18	0.419	0.299	0.299–1.568	0.683	2	3.290	3.290	0.300–6.270	0.648	16	11.580	1.410	0.300–90.280	0.465	10	3.250	2.430	0.300–12.910	0.644
2 a 5	11	0.299	0.299	0.299–0.299		41	0.652	0.299	0.299–2.936		16	3.860	0.300	0.300–29.450		30	9.330	1.620	0.300–83.270		13	1.745	2.195	0.299–3.859	
>5	71	0.587	0.299	0.299–4.796		128	0.646	0.299	0.299–11.848		190	2.967	0.299	0.299–37.069		249	7.780	0.300	0.300–95.810		206	2.685	1.236	0.299–23.138	
Alcohol																									
Consumers	41	0.487	0.299	0.299–3.767	0.814	98	0.683	0.299	0.299–11.848	0.403	96	3.204	0.299	0.299–35.784	0.893	116.000	9.530	0.300	0.300–95.810	0.887	77	2.362	1.122	0.299–21.638	0.362
Non-consumers	39	0.566	0.299	0.299–4.796		75	0.473	0.299	0.299–5.279		50	2.980	0.299	0.299–24.044		119.000	5.790	0.340	0.300–73.280		71	2.932	1.177	0.299–22.080	
Smoking habit																									
Smokers	7	1.657	0.299	0.299–4.796		27	0.463	0.299	0.299–2.111	0.834	13	3.030	0.300	0.300–10.340	0.643	61.000	4.720	0.510	0.300–67.280	0.909	14	1.783	0.299	0.299–6.443	0.213
Nonsmokers	73	0.417	0.299	0.299–3.767	0.108	146	0.616	0.299	0.299–11.847		131	3.105	0.299	0.299–35.784		176.000	8.580	0.300	0.300–95.810		135	2.839	1.314	0.299–22.080	
Drinking water source																									
Well	87	0.534	0.299	0.299–4.796	*	120	0.516	0.299	0.299–11.848	0.002	114	3.407	0.299	0.299–37.069	0.395	215	8.640	0.320	0.300–95.810	0.299	100	3.045	1.432	0.299–23.138	0.966
General network	*	*	*	*		67	0.821	0.299	0.299–4.772		92	2.510	0.299	0.299–29.447		80	6.810	0.300	0.300–86.270		125	2.411	1.346	0.299–19.085	
River	*	*	*	*		*	*	*	*		*	*	*	*		*	*	*	*		*	*	*	*	

AM—arithmetic mean; MED—median; * Does not present any individual in the studied group; ** Comparisons between the studied variables: *p* ≤ 0.05.

**Table 6:** The description of the statistical analysis by location (Group 2) with higher levels of Ba (µg·L^−1^) by the variables studied.

GROUPS	BCA					VBJ					DMN					CUR					ISJ				
	N	AM	MED	RANGE	*p* **	N	AM	MED	RANGE	*p* **	N	AM	MED	RANGE	*p* **	N	AM	MED	RANGE	*p* **	N	AM	MED	RANGE	*p* **
Gender																									
Female	38	10.810	8.700	0.300–45.490	0.762	162	12.467	12.035	0.299–42.839	0.113	19	9.127	8.869	7.463–11.378	0.168	9	5.000	8.510	1.980–25.260	0.009	12	28.760	25.220	1.20–64.780	0.204
Male	36	11.210	10.750	0.300–34.780		132	13.933	14.367	0.299–50.740		23	8.592	8.604	7.156–10.617		12	19.080	19.450	3.520–32.640		18	39.320	41.060	3.480–84.610	
Age group (years)																									
0 a 10	23	12.500	9.570	0.300–40.820	0.935	52	11.474	10.727	0.299–36.010	0.577	11	8.758	8.604	7.669–10.438	0.041	4	17.210	17.540	4.100–31.650	0.818	8	41.200	44.900	6.700–84.600	0.117
11 a 19	10	10.310	5.930	0.300–45.490		59	13.144	14.826	0.299–25.463		9	9.281	9.786	7.542–10.617		2	15.000	15.000	4.800–25.300		7	39.570	37.610	15.710–60.270	
20 a 59	36	10.570	8.530	0.300–34.780		140	13.533	14.082	0.299–50.740		18	8.954	8.825	7.558–11.378		14	12.520	13.670	1.980–32.640		13	33.960	27.800	7.510–67.680	
≥60	5	8.650	11.560	2.78–13.640		43	13.770	15.030	0.300–42.840		4	7.497	7.434	7.156–7.965		1	18.357	18.357	18.357–18.357		2	2.340	2.340	1.200–3.480	
Residence time (years)																									
<2	6	16.860	11.370	0.300–34.780	0.314	14	14.930	14.970	0.300–36.300	0.571	5	8.959	8.74	7.669–10.617	0.713	*	*	*	*	0.713	8	38.510	40.840	1.200–69.580	0.741
2 a 5	39	12.200	8.540	0.300–45.490		36	12.040	10.010	0.300–38.210		4	9.15	9.112	7.938–10.438		1	19.852	19.852	19.852–19.852		3	46.000	45.200	8.000–84.600	
>5	29	8.180	6.060	0.300–22.940		244	13.181	13.982	0.299–50.740		33	8.777	8.604	7.156–11.378		20	13.660	14.510	1.980–32.640		19	31.940	27.090	3.480–64.780	
Alcohol																									
Consumers	41	0.487	0.299	0.299–3.767	0.814	179	12.555	12.531	0.299–42.839	0.313	10	9.019	8.691	7.156–11.378	0.429	8	10.530	13.410	1.980–31.650	0.515	17	37.180	44.380	1.200–84.610	0.688
Non-consumers	39	0.566	0.299	0.299–4.796		115	14.013	14.333	0.299–50.740		12	8.415	8.024	7.405–9.686		13	13.920	15.590	3.520–32.640		10	32.030	27.450	7.510–67.680	
Smoking habit																									
Smokers	10	10.130	3.880	0.300–29.930	0.892	49	14.190	14.980	0.300–29.800	0.179	6	8.063	7.987	7.405–9.362	0.106	5	14.560	14.960	5.000–29.810	0.804	1	21.308	21.308	21.308–21.308	0.108
Nonsmokers	63	11.230	9.870	0.300–45.490		245	12.913	12.334	0.299–50.74		36	8.963	8.825	7.156–11.378		16	13.660	14.700	1.980–32.640		26	35.810	37.450	1.200–84.610	
Drinking water source																									
Well	74	11.010	9.210	0.300–45.490	*	156	12.959	13.381	0.299–36.297	0.902	42	8.834	8.672	7.156–11.378	*	20	13.660	14.580	1.980–32.640	*	*	*	*	*	*
General network	*	*	*	*		138	13.313	13.354	0.299–50.740		*	*	*	*		*	*	*	*		*	*	*	*	
River	*	*	*	*		*	*	*	*		*	*	*	*		*	*	*	*		30	35.100	36.010	1.200–84.610	

AM—arithmetic mean; MED—median; * Does not present any individual in the studied group; ** Comparisons between the studied variables: *p* ≤ 0.05.

**Table 7:** Statistical analysis of eating habits by localities (Group 1) with lower blood Ba levels (µg·L^−1^) by the epidemiological variables.

EATING HABITS (WEEK)	ACUI					BIN					VIT					CLA					VCO				
	N	MA	MED	RANGE	*p* **	N	MA	MED	RANGE	*p* **	N	MA	MED	RANGE	*p* **	N	MA	MED	RANGE	*p* **	N	MA	MED	RANGE	*p* **
Chicken																									
≤1	6	0.299	0.299	0.299–0.299	0.728	13	0.543	0.299	0.299–2.506	0.761	40	3.220	0.300	0.3–31.220	0.702	30	6.810	0.540	0.300–66.170	0.772	30	3.346	1.664	0.299–22.080	0.452
2 A 4	65	0.587	0.299	0.299–4.796		126	0.686	0.299	0.199–11.848		144	3.131	0.299	0.299–37.069		227	7.610	0.320	0.300–95.810		176	2.534	1.236	0.299–23.138	
>4	16	0.407	0.299	0.299–2.029		48	0.473	0.299	0.299–2.748		24	2.150	0.300	0.300–29.450		38	10.030	0.300	0.300–68.990		23	2.593	1.527	0.299–11.459	
Meat																									
≤1	24	0.521	0.299	0.299–3.767	0.619	21	0.304	0.299	0.299–0.405	0.280	32	1.928	0.299	0.299–10.601	0.919	43	5.710	0.700	0.300–66.170	0.368	22	4.281	2.941	0.299–19.085	0.005
2 A 4	61	0.511	0.299	0.299–4.795		107	0.674	0.299	0.299–11.848		140	3.015	0.299	0.299–37.069		220	8.390	0.300	0.300–95.810		182	2.613	1.166	0.299–23.138	
>4	2	1.400	1.400	0.3–2.49		59	0.639	0.299	0.199–2.789		36	4.100	0.300	0.300–32.550		32	6.880	0.730	0.300–68.990		25	1.450	0.824	0.299–7.007	
Fish																									
≤1	29	0.658	0.299	0.299–4.796	0.899	107	0.700	0.299	0.199–11.848	0.777	119	2.944	0.299	0.299–35.784	0.814	169	7.020	0.310	0.300–95.810	0.034	105	2.108	0.824	0.299–22.08	0.020
2 A 4	54	0.485	0.299	0.299–3.365		72	0.514	0.299	0.299–2.748		81	3.324	0.299	0.299–37.069		101	7.750	0.300	0.300–86.270		117	2.992	1.683	0.299–23.138	
>4	4	0.299	0.299	0.299–0.299		8	0.533	0.299	0.299–1.199		8	1.466	0.299	0.299–5.069		25	13.750	2.560	0.300–68.990		7	4.950	3.160	1.350–17.590	
Brazil nuts																									
≤1	52	0.565	0.299	0.299–4.796	0.987	175	0.545	0.299	0.199–5.279	0.109	203	2.774	0.299	0.299–35.784	0.035	282	8.070	0.310	0.300–95.810	0.692	226	2.647	1.298	0.299–23.138	0.830
2 A 4	17	0.446	0.299	0.299–2.348		11	1.870	0.910	0.300–11.850		3	21.960	24.040	4.770–37.070		10	3.620	0.300	0.300–26.670		3	2.640	1.520	0.300–6.100	
>4	18	0.529	0.299	0.299–2.495		1	0.299	0.299	0.299–0.299		2	1.174	1.174	0.299–2.049		3	0.378	0.320	0.299–0.514		*	*	*	*	
Shellfish and crustaceans																									
≤1	79	0.558	0.299	0.299–4.796	0.518	168	0.626	0.299	0.199–11.847	0.906	165	3.125	0.299	0.299–37.069	0.473	271	8.070	0.300	0.300–95.810	0.185	175	2.812	1.625	0.299–22.08	0.010
2 A 4	8	0.299	0.299	0.299–0.299		12	0.586	0.299	0.299–2.748		26	3.350	0.299	0.299–21.080		13	3.460	0.300	0.300–26.670		48	1.863	0.299	0.299–23.138	
>4	*	*	*	*		7	0.567	0.299	0.299–1.631		17	1.682	0.299	0.299–7.718		11	7.310	2.400	0.300–53.240		6	4.090	1.800	0.530–12.810	
Vegetable																									
≤1	29	0.776	0.299	0.299–4.796	0.632	62	0.699	0.299	0.299–11.848	0.902	52	2.086	0.299	0.299–26.198	0.560	47	6.790	0.300	0.300–86.270	0.306	61	3.791	2.058	0.299–23.138	0.031
2 A 4	44	0.449	0.299	0.299–3.365		89	0.610	0.299	0.199–4.7723		123	2.863	0.299	0.299–35.784		130	7.670	0.720	0.300–83.270		122	2.150	0.909	0.299–22.080	
>4	14	0.299	0.299	0.299–0.299		36	0.516	0.299	0.299–1.7012		33	5.170	0.300	0.300–37.070		118	8.440	0.430	0.300–95.810		46	2.445	1.537	0.299–12.812	
Fruit									-																
≤1	87	0.534	0.299	0.299–4.7959	*	187	0.621	0.299	0.199–11.847	*	63	2.353	0.299	0.299–19.527	0.123	40	5.830	0.300	0.300–66.170	0.224	53	3.724	1.973	0.299–23.138	0.020
2 A 4	*	*	*	*		*	*	*	*		55	5.050	0.300	0.300–37.070		147	7.630	0.300	0.300–86.270		67	1.878	0.608	0.299–12.812	
>4	*	*	*	*		*	*	*	*		90	2.280	0.299	0.299–32.548		108	8.860	0.680	0.300–95.810		109	2.595	1.122	0.299–22.080	

AM—arithmetic mean; MED—median; * Does not present any individual in the studied group; ** Comparisons between the studied variables: *p* ≤ 0.05.

**Table 8:** Statistical analysis of eating habits by localities (Group 2) with lower blood Ba levels (µg·L^−1^) by the epidemiological variables.

EATING HABITS (WEEK)	BCA					VBJ					DMN					CUR					ISJ				
	N	MA	MED	RANGE	*p*	N	MA	MED	RANGE	*p* **	N	MA	MED	RANGE	*p* **	N	MA	MED	RANGE	*p* **	N	MA	MED	RANGE	*p* **
Chicken																									
≤1	2	9.940	9.940	0.300–19.580	0.828	58	12.080	12.180	0.300–42.840	0.462	10	8.781	8.611	7.665–10.773	0.984	1	18.357	18.357	18.357–18.357	0.620	14	34.330	34.510	3.480–64.780	0.901
2 A 4	72	11.030	9.210	0.300–45.490		216	13.392	12.964	0.299–50.740		28	8.878	8.671	7.156–11.378		20	13.660	14.580	1.980–32.640		16	35.770	36.010	1.200–84.6100	
>4	*	*	*			20	13.140	14.170	0.300–24.150		4	8.656	8.782	7.558–9.503		*	*	*	*		*	*	*	*	
Meat																									
≤1	8	11.420	5.800	0.300–33.770	0.629	34	10.670	8.100	0.300–28.530	0.087	19	9.139	9.251	7.542–11.378	0.132	5	16.270	15.280	7.070–19.850	0.620	14	34.330	34.510	3.480–64.780	0.901
2 A 4	65	11.100	9.870	0.300–45.490		237	13.244	14.001	0.299–42.839		22	8.636	8.603	7.156–10.947		16	12.780	14.600	1.980–32.640		16	35.770	36.010	1.200–84.610	
>4	1	1.408	1.408	1.408–1.408		23	15.420	16.320	0.300–50.740		1	7.405	7.405	7.405–7.405		*	*	*	*		*	*	*	*	
Fish																									
≤1	36	12.150	10.110	0.30–45.490	0.192	109	12.717	13.542	0.299–28.427	0.219	28	8.703	8.603	7.542–10.773	0.631	17	13.920	15.000	1.980–32.640	0.796	*	*	*	*	0.553
2 A 4	34	10.670	9.360	0.300–33.770		174	12.934	12.438	0.299–50.740		14	9.096	9.184	7.156–11.378		3	13.410	12.250	3.520–19.810		18	33.580	31.260	1.200–84.610	
>4	4	3.580	2.220	0.300–9.570		11	19.960	18.280	0.300–42.790		*	*	*	*		1	18.357	18.357	18.357–18.357		12	37.370	43.900	6.670–64.780	
Brasil nuts																									
≤1	57	12.210	9.870	0.300–45.490	0.445	252	12.988	12.546	0.299–50.74	0.052	42	8.834	8.672	7.156–11.378	*	8	17.310	16.200	50.000–29.810	0.339	27	33.700	34.730	1.200–84.610	0.284
2 A 4	12	6.980	5.170	0.300–15.220		35	14.970	15.090	0.300–42.840		*	*	*	*		1	3.519	3.519	3.519–3.519		*	*	*		
>4	5	6.950	5.330	0.300–14.200		7	8.490	5.040	0.300–38.210		*	*	*	*		12	12.780	14.740	1.980–32.640		3	47.700	64.800	8.700–69.600	
Shellfish and crustaceans																									
≤1	53	11.630	9.570	0.300–45.490	0.425	239	12.866	12.762	0.299–42.839	0.542	42	8.834	8.672	7.156–11.378	*	19	13.920	15.160	1.980–32.640	0.549	19	30.820	27.800	3.480–69.580	0.468
2 A 4	21	9.430	5.330	0.300–29.930		55	14.210	14.170	0.300–50.740		*	*	*	*		2	10.940	10.940	3.520–18.360		6	41.600	44.100	1.200–84.600	
>4	*	*	*	*							*	*	*	*		*	*	*	*		5	43.500	60.300	8.700–64.800	
Vegetable																									
≤1	8	12.550	13.560	0.300–29.780	0.975	62	14.047	14.900	0.299–42.839	0.201	5	8.337	8.604	7.156–9.503	0.540	1	29.812	29.812	29.812–29.812	0.409	14	30.250	25.870	3.480–64.780	0.582
2 A 4	13	11.000	6.060	0.300–40.820		191	13.207	13.166	0.299–50.740		24	9.005	8.825	7.405–11.378		13	13.410	13.760	1.980–32.640		11	41.990	44.520	1.200–84.610	
>4	53	10.770	9.570	0.300–45.490		41	11.290	8.330	0.300–31.050		13	8.710	8.124	7.542–10.617		7	16.270	14.480	3.400–31.650		5	33.510	37.300	13.890–50.960	
Fruit																									
≤1	74	11.000	9.210	0.300–45.490	*	41	11.980	8.330	0.300–28.430	0.018	10	8.245	8.238	7.156–9.131	0.315	21	13.920	14.760	1.980–32.640	*	30	35.100	36.010	1.200–84.610	*
2 A 4	*	*	*	*		185	14.181	14.932	0.299–50.740		23	9.029	8.869	7.405–11.378		*	*	*	*		*	*	*	*	
>4	*	*	*	*		68	10.905	9.149	0.299–31.045		9	8.990	9.117	7.750–10.947		*	*	*	*		*	*	*	*	

AM—arithmetic mean; MED—median; * Does not present any individual in the studied group; ** Comparisons between the studied variables: *p* ≤ 0.05.

**Table 9:** The correlation test and linear regressions of Ba concentrations in relation to the independent variables.

Variable	r_s_	*p*-Value	R^2^ _adj_	*p* Value
Proximity to impacted rivers	0.018	0.500	0.0	0.310
Proximity to the kaolin industry	0.144	<0.001	1.6	<0.001
Water consumption	0.103	<0.001	2.2	<0.001
Weekly consumption of fish	0.089	<0.001	1.5	<0.001

## Data Availability

Available on request from the corresponding author due to some of this data is part of an ongoing study.
